# Identification and Expression Profiling of Toll-Like Receptors of Brown Trout (*Salmo trutta*) during Proliferative Kidney Disease

**DOI:** 10.3390/ijms21113755

**Published:** 2020-05-26

**Authors:** Arun Sudhagar, Mansour El-Matbouli, Gokhlesh Kumar

**Affiliations:** 1 Clinical Division of Fish Medicine, University of Veterinary Medicine, 1210 Vienna, Austria; Arun.Sudhagar@vetmeduni.ac.at (A.S.); Mansour.El-Matbouli@vetmeduni.ac.at (M.E.-M.); 2 Central Institute of Fisheries Education, Rohtak Centre, Haryana 124411, India

**Keywords:** pattern recognition receptors, TLRs, *Tetracapsuloides bryosalmonae*, myxozoan, parasite

## Abstract

Proliferative kidney disease is an emerging disease among salmonids in Europe and North America caused by the myxozoan parasite *Tetracapsuloides bryosalmonae.* The decline of endemic brown trout (*Salmo trutta*) in the Alpine streams of Europe is fostered by *T. bryosalmonae* infection. Toll-like receptors (TLRs) are a family of pattern recognition receptors that acts as sentinels of the immune system against the invading pathogens. However, little is known about the TLRs’ response in salmonids against the myxozoan infection. In the present study, we identified and evaluated TLR1, TLR19, and TLR13-like genes of brown trout using data-mining and phylogenetic analysis. The expression pattern of TLRs was examined in the posterior kidney of brown trout infected with *T. bryosalmonae* at various time points. Typical Toll/interleukin-1 receptor protein domain was found in all tested TLRs. However, TLR13-like chr2 had a short amino acid sequence with no LRR domain. Phylogenetic analysis illustrated that TLR orthologs are conserved across vertebrates. Similarly, a conserved synteny gene block arrangement was observed in the case of TLR1 and TLR19 across fish species. Interestingly, all tested TLRs showed their maximal relative expression from 6 to 10 weeks post-exposure to the parasite. Our results suggest that these TLRs may play an important role in the innate defense mechanism of brown trout against the invading *T. bryosalmonae*.

## 1. Introduction

*Tetracapsuloides bryosalmonae,* a cnidarian myxozoan endoparasite belonging to the class malacosporea is responsible for causing proliferative kidney disease (PKD) in salmonids [1]. This parasite is widespread across the continents of Europe and North America, and it causes massive economic losses to the salmonid farming industry [2]. Moreover, *T. bryosalmonae* threatens the wild salmonid population [3]. *T. bryosalmonae* infection along with the climate-change driven increase of water temperature in the Alpine streams is responsible for the population decline of native brown trout (*Salmo trutta*) [4,5]. In the summer of 2016, an outbreak of *T. bryosalmonae* infection at the Yellowstone river in the state of Montana, USA resulted in the mass mortality of salmonids, predominantly mountain whitefish (*Prosopium williamsoni*) [6]. This incident led to the temporary emergency closure of a section of the river to the public, resulting in a significant economic impact on the local tourism and recreational industry [7].

*T. bryosalmonae* has a two-host life cycle involving a bryozoan and a salmonid fish host [8,9]. The *T. bryosalmonae* spores released by bryozoans utilize gills for their portal entry into the salmonid host and migrate to various internal organs including kidney, spleen, and liver [10]. However, the kidney is the primary organ for sporogenesis of *T. bryosalmonae*, and after maturation in the kidney, the parasite is shed via urine [10,11]. The extra-sporogonic development of *T. bryosalmonae* in the interstitial tissues of the kidney leads to an inflammatory response, which results in the swelling of the kidney [12,13,14]. Currently, there are no practical treatments or preventive measures available for *T. bryosalmonae*. Interestingly, in Europe, co-evolution was observed between *T. bryosalmonae* (European lineage) and native trout species such as brown trout and brook trout (*Salvelinus fontinalis*). Nevertheless, the parasite did not co-evolve with the North American rainbow trout (*Oncorhynchus mykiss*). *T. bryosalmonae*-infected brown trout and brook trout could successfully transmit the parasite to the bryozoan via urine; however, this transmission was not possible from the infected rainbow trout host [8,9,15]. Furthermore, PKD recovered brown trout can shed parasitic spores even after five years post-infection [16]. Immune responses of rainbow trout during PKD have been studied at the molecular level. PKD-infected rainbow trout exhibits high antibody response, the up-regulation of suppressors of cytokine signaling molecules, dysregulated B cell populations, and cytokines of T-helper cells [17,18,19,20,21,22,23]. Nevertheless, only a few experiments have been conducted in brown trout to understand the host immune responses [22,24,25], protein–protein interaction [26], and metabolic functions [27] during PKD pathogenesis.

Toll-like receptors (TLRs) are a class of proteins belonging to the pattern recognition receptors (PRRs) of the host that recognize a wide range of conserved pathogen-associated molecular patterns (PAMPs) of the pathogens. TLRs serve as the front line of host defense response against infecting pathogens by activating an intracellular signaling cascade to elicit an early inflammatory and innate immune response [28]. TLRs have three domains such as extracellular, transmembrane (TM), and intracellular signaling domains. The extracellular domain of TLRs have leucine-rich repeats (LRRs) which recognizes PAMPs, whereas the cytoplasmic domain is similar to interleukin-1 (IL-1) receptors and hence named as the Toll/interleukin-1 receptor (TIR) domain. The TIR domain is responsible for cell signaling [29]. Parasites can strategically evade their host by manipulating the host immune action and understanding this mechanism during host–parasite interaction is indispensable for developing effective prophylaxis and treatments. TLRs are well known for eliciting an innate immune action against protozoan and metazoan parasitic infections in higher vertebrates [30,31]. Similarly, parasitic infections are known to modulate TLRs in fish during *Dactylogyrus intermedius* [32] and *Ichthyophthirius multifiliis* infections [33]. In a brief experiment, TLRs were up-regulated (TLR8a1, TLR8a2, TLR8b1, TLR19, TLR20a, TLR22a1, and TLR22a2) and down-regulated (TLR3, TLR8b2, TLR9, TLR18, and TLR21) in the kidney of rainbow trout during natural PKD infection [34]. Moreover, intraperitoneal injection of live theront life stages of *I. multifiliis* modulated the expression of TLRs in channel catfish (*Ictalurus punctatus*) [35].

In our recent transcriptome study, we identified three differentially expressed TLRs (TLR1, TLR19, and TLR13-like) in the kidney of brown trout during *T. bryosalmonae* development [25]. TLR1 is found in mammals belonging to the TLR1 subfamily, whereas TLR13 and TLR19 belong to the TLR11 subfamily [36]. However, there is no detailed information regarding TLRs and their functions in brown trout during PKD pathogenesis. Hence, the present investigation was aimed to analyze TLR1, TLR19, and four TLR13-like genes, and to perform a time-series investigation in the expression pattern of these TLRs during PKD pathogenesis in brown trout.

## 2. Results

### 2.1. Brown Trout Infection

**Figure 1:**
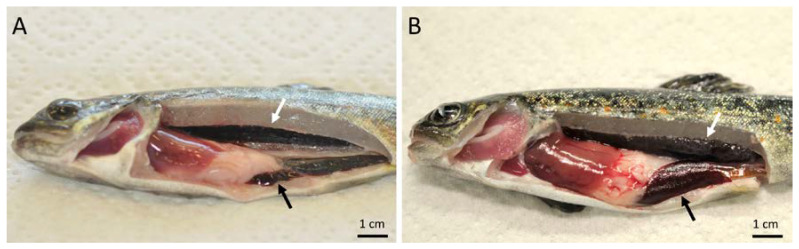
Brown trout exhibiting clinical signs of proliferative kidney disease. (A) Unexposed control brown trout having normal spleen (black arrow) and kidney (white arrow). (**B**) Exposed brown trout showing swollen spleen (black arrow), and swollen kidney (white arrow).

**Figure 2:**
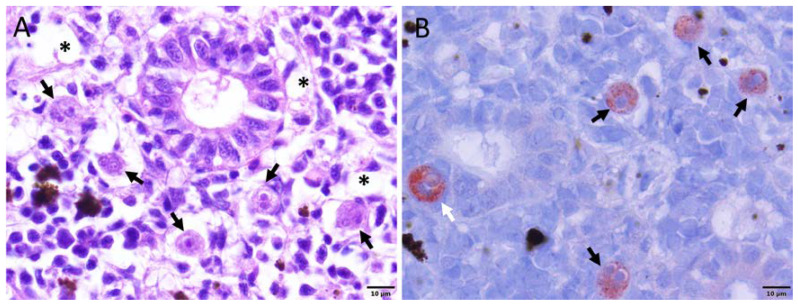
Histopathology and immunohistochemistry of kidney sections. (A) Kidney showing renal degeneration (asterisk) and pre-sporogonic stages of the parasite (black arrows) in the renal interstitium. (**B**) Pre-sporogonic (black arrows) and interepithelial stages of the parasite (white arrow). This section was stained using immunohistochemistry and a hematoxylin counter-stain.

During the initial time point of sampling, 2 weeks post-exposure (wpe), no clinical signs of the disease were observed in the exposed group. At 4 wpe, only one fish had a slightly swollen kidney and spleen. At 6, 8, 10, and 12 wpe, fish showed typical clinical signs of PKD such as renal hyperplasia, splenomegaly, and pale liver (Figure 1). However, no clinical signs of PKD were observed in the exposed group at 17 wpe. Similarly, no fish from the control group showed any clinical signs of the disease throughout the experiment. The histological observation of exposed brown trout exhibited the degeneration of kidney tubules, necrosis, and decrease of melanomacrophages in the kidney (Figure 2A). Moreover, *T. bryosalmonae* stages were observed in the kidney of exposed brown trout from 4–12 wpe using immunohistochemistry (IHC) (Figure 2B). However, no *T. bryosalmonae* stage was observed in the kidney at 2 and 17 wpe.

### 2.2. Identification of Selected TLRS in Brown Trout

mRNA sequence, amino acid sequence, and transcriptional direction were identified for all the selected TLRs from the brown trout genome (Table 1). TLR1 and TLR19 had a single copy and were located on chromosome 3. Whereas, TLR13-like had multiple copies and the transcript of TLR13-like located in chromosome 1 (TLR13-like Chr1) had 99.76% identity with the input sequence [25]. Each TLR gene had a complete open reading frame ranging between 322 (TLR13-like Chr2) and 1027 (TLR13-like Chr1) amino acids. Among the analyzed genes, TLR13-like Chr2 had the shortest length with 1422 nucleotides and 322 amino acids. Theoretical isoelectric point (pI) values were in the range between 6.01 (TLR13-like Chr1) and 7.32 (TLR13-like Chr6) and the predicted molecular weight (MW) were in the range of 38.4 kDa (TLR13-like Chr2) to 117.4 kDa (TLR13-like Chr1). Subcellular location predicted TLR1, TLR19, and TLR13-like Chr2 proteins on the plasma membrane. Moreover, other brown trout TLR proteins were localized on the membrane-bound organelles such as endoplasmic reticulum (TLR13-like Chr1), mitochondria (TLR13-like Chr6), and vacuoles (TLR13-like Chr27).

### 2.3. TLR Domain Analysis

**Figure 3:**
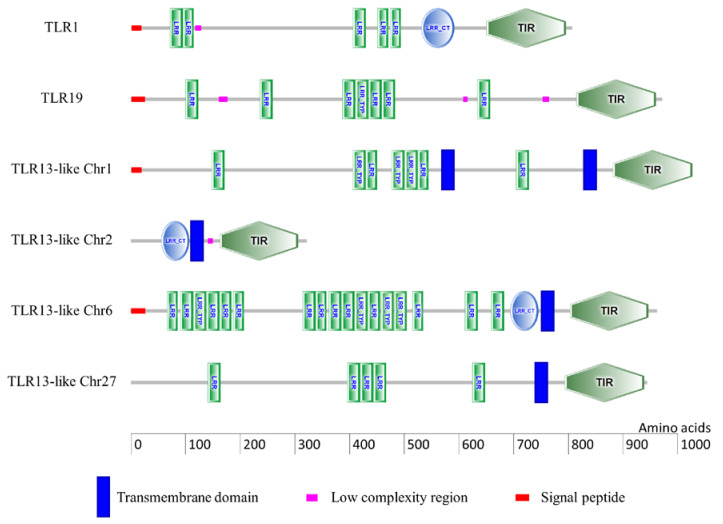
Schematic representation of the domain architecture of six TLRs of brown trout used in the present study. The domain architectures were predicted by the SMART tool. The domain abbreviations are as follows: LRR—Leucine-rich repeat; LRR-CT—LRR C-terminal; LRR TYP—typical LRR; TIR—Toll/IL-1 receptor.

Typical conserved functional domains such as the LRR domain, TIR domain, and TM domain were observed in the predicted TLR proteins (Figure 3). In each predicted TLR protein, the number of LRR domains differed. For instance, TLR1, TLR19, TLR13-like Chr1, TLR13-like Chr6, and TLR13-like Chr27 had 5, 7, 7, 17, and 5 LRR domains, respectively. However, TLR13-like Chr2 was shorter in length and did not have any LRR domain. Similarly, TLR1, TLR13-like Chr2, and TLR13-like Chr6 had one LRR C-terminal (LRR-CT) domain each. TM domain was absent in TLR1 and TLR19, whereas all four TLR13-like proteins had a TM domain. A putative signal peptide was observed in the N-terminal region of TLR1 (1–19 amino acids), TLR19 (1–26 amino acids), TLR13-like Chr1 (1–20 amino acids), and TLR13-like Chr6 (1–27 amino acids).

### 2.4. Synteny Analysis

**Figure 4:**
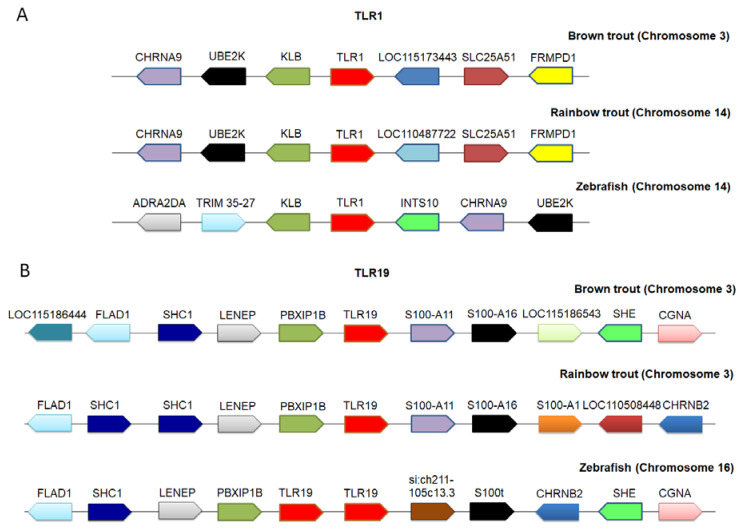
Comparative synteny analysis of TLRs. The analysis of TLRs was performed using the Genomicus online database, NCBI, and ENSEMBL genome database. The arrows indicate the transcriptional direction and the same genes are represented in similar color across species. (**A**) TLR1 synteny arrangement (brown trout chromosome 3—NC_042959.1; rainbow trout chromosome 14—NC_035090.1; zebrafish chromosome 14—genomicus database); (**B**) TLR19 synteny arrangement (brown trout chromosome 3—NC_042959.1; rainbow trout chromosome 3—NC_035079.1; zebrafish chromosome 16—genomicus database). The full names of gene symbols are provided in Supplementary Table S1.

In comparative genomics, local synteny analysis is one of the effective methods to identify orthologous genes across species [39]. Based on our synteny analysis, TLR6-like (XM_029731478.1 and XP_029587338.1) and TLR12-like (XM_029746142.1 and XP_029602002.1) genes were re-annotated as TLR1 and TLR19, respectively based on the gene nomenclature of model organism zebrafish (*Danio rerio*). Conserved synteny was found among brown trout, rainbow trout, and zebrafish in case of TLR1, with neighboring genes KLB, UBE2K, CHRNA9, SLC25A51, and FRMPD1 (Figure 4A). Likewise, highly conserved syntenic arrangement was found around TLR19 of brown trout, rainbow trout, and zebrafish with PBXIP1B, LENEP, SCH1, FLAD1, S100-A11, S100-A16 genes, SHE, and GCNA (Figure 4B). The upstream and downstream synteny gene blocks to TLRs were conserved across fish species.

### 2.5. Phylogenetic Analysis

**Figure 5:**
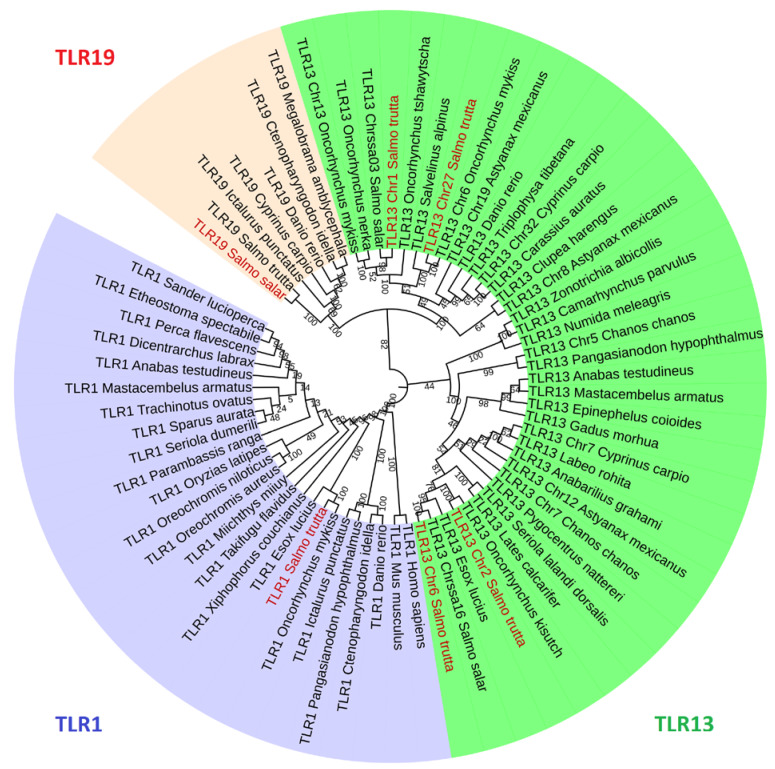
Phylogenetic tree of TLRs from different vertebrates. The tree was generated using MEGA X using the maximum likelihood method. The different gene groups are denoted by various colors, TLR1—blue; TLR19—red; TLR13-like—dark green. The leaves with red legend indicate brown trout TLRs. The GenBank accession numbers of the sequences are available in Supplementary Table S2.

All the TLR proteins shared the closest relationship with the corresponding orthologs from other vertebrates including distant species such as human (*Homo sapiens*) and mouse (*Mus musculus*) in case of TLR1; small tree finch (*Camarhynchus parvulus*), white-throated sparrow (*Zonotrichia albicollis*), and helmeted guineafowl (*Numida meleagris*) for the TLR13-like gene. This shows that the TLR orthologs are evolutionarily conserved across species. TLR1 belonging to the TLR1 subfamily emerged as a separate monophyletic clade, whereas TLR19 and some TLR13-like genes categorized under the TLR11 subfamily branched as a separate clade. Among the brown trout genes belonging to the TLR11 subfamily, TLR19, TLR13-like Chr1, and TLR13-like Chr27 are closely related, whereas TLR13-like Chr2 and TLR13-like Chr6 emerged in a separate clade. The phylogenetic analysis showed that the brown trout TLRs are closely related with other salmonid TLRs such as Atlantic salmon (*Salmo salar*), coho salmon (*Oncorhynchus kisutch*), rainbow trout, Chinook salmon (*Oncorhynchus tshawytscha*), sockeye salmon (*Oncorhynchus nerka*), and Arctic charr (*Salvelinus alpinus*) (Figure 5).

### 2.6. Time Series Expression Analysis of TLRs during Proliferative Kidney Disease

**Figure 6:**
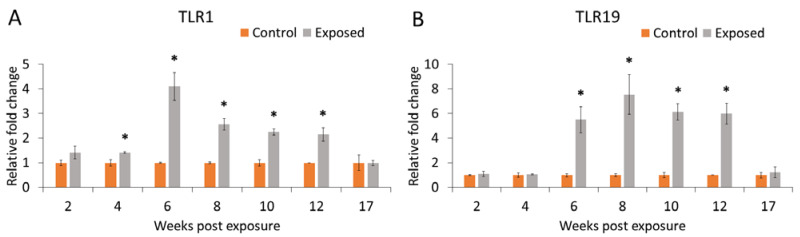
Relative expression of TLR1 and TLR19 in the posterior kidney of brown trout. (**A**) Expression profile of TLR1 that attained a peak at 6 wpe. (**B**) Expression profile of TLR19, which had a peak expression at 8 wpe. Significant differences in the mRNA expression of each gene between the exposed and control groups at each time points are indicated with an * (*p* < 0.05). Each bar shows the mean ± SEM (*n* = 9).

The expression of TLR1 and TLR19 was slightly up-regulated in the kidney of exposed groups at 2 wpe, but it was non-significant compared to control groups. At 6 wpe, the relative expression of TLR1 attained a peak (4.1 fold) and then gradually reduced and became normal in the kidney of exposed brown trout at 17 wpe (Figure 6A). There was no significant difference in the expression of TLR19 in the kidney of exposed and control groups during initial time points at 2 and 4 wpe. However, there was a 5.5 fold up-regulation of TLR19 at 6 wpe, which further increased to 7.5 fold at 8 wpe. The expression of TLR19 was gradually reduced in the kidney at 10 wpe (6.1 fold) and 12 wpe (5.9 fold) and then became normal at 17 wpe (Figure 6B).

**Figure 7:**
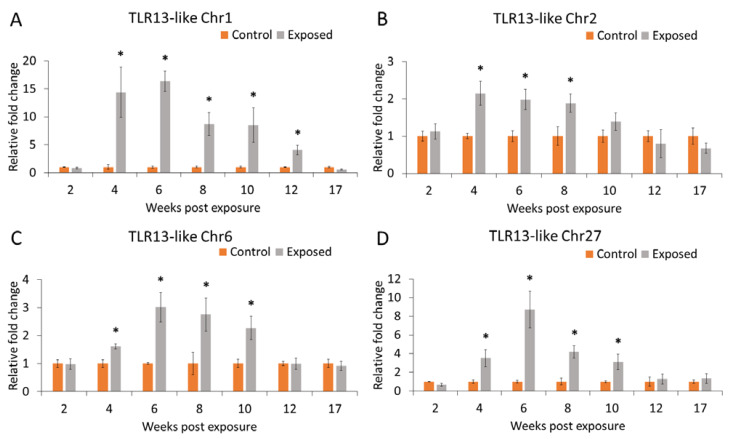
Relative expression of TLR13-like genes in the posterior kidney of brown trout. (**A**) Expression profile of TLR13-like Chr1. (**B**) Expression pattern of TLR13-like Chr2. (**C**) Expression pattern of TLR13-like Chr6. (**D**) Expression pattern of TLR13-like Chr27. Significant differences in the mRNA expression between the exposed and control groups at each time points are indicated with an * (*p* < 0.05). Each bar shows the mean ± SEM (*n* = 9).

**Figure 8:**
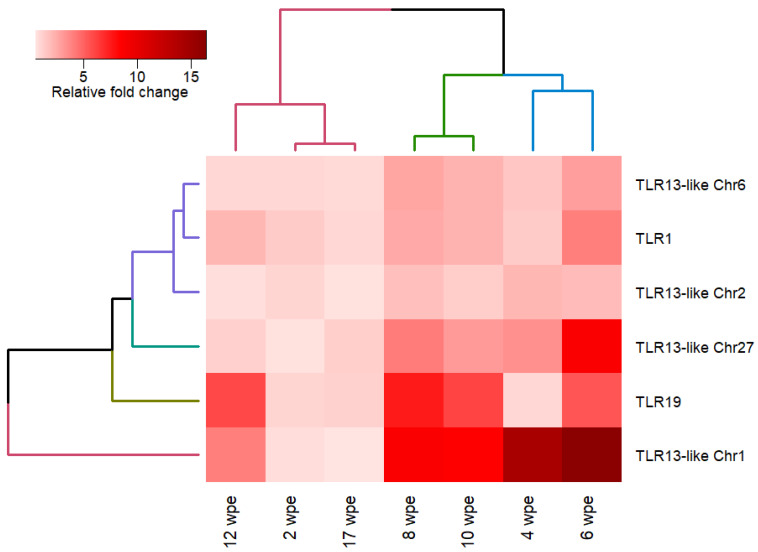
Heatmap representation of quantitative real time (qRT-PCR) expression profile of TLRs in the posterior kidney of brown trout at various time points. The expression levels of genes are presented using relative fold change values of exposed compared to control groups. Euclidean distance and complete linkage method was used for hierarchical clustering.

TLR13-like Chr1 was significantly up-regulated in the kidney at all time points except 2 and 17 wpe. A strong up-regulation of TLR13-like Chr1 was noticed at 6 wpe (16.3 fold). Similarly, TLR13-like Chr27 was up-regulated at 4–10 wpe with a peak at 6 wpe (8.7 fold). However, TLR13-like Chr2 and TLR13-like Chr6 had a moderate up-regulation at 6–10 wpe. Overall, the expression of all tested TLRs except TLR13-like Chr27 was non-significantly down-regulated at 17 wpe during PKD pathogenesis (Figure 7). There was no significant difference in the expression pattern of all the examined TLRs in the kidney of exposed and control groups at 2 wpe and 17 wpe, and these two time points were clustered together in hierarchical clustering (Figure 8).

## 3. Discussion

The innate immune system can detect conserved molecular signatures of invading pathogens called PAMPs with the help of the host components called PRRs. TLRs belong to one such family of PRR that can recognize PAMPs and activate the immune system against the invading pathogens. The number of TLRs varies between species, and different TLRs can detect different PAMPs and thereby helps the immune system to recognize a wide range of invading pathogens. The TLRs are broadly classified under six subfamilies: the TLR1 subfamily (TLR1, TLR2, TLR6, TLR10, TLR14, TLR15, TLR16, TLR18, TLR25, TLR27, and TLR28), TLR3 subfamily (TLR3), TLR4 subfamily (TLR4), TLR5 subfamily (TLR5), TLR7 subfamily (TLR7, TLR8, and TLR9), and TLR11 subfamily (TLR11, TLR19, TLR13, TLR19–23, and TLR26) [36]. RNA-seq based transcriptome data are valuable resources for mining and studying specific family of genes from non-model species [40,41]. Moreover, transcriptome data serve as a valuable baseline resource to explore both host–parasite relationships [42]. Three TLRs (TLR1, TLR19, and TLR13-like) were differentially expressed in the kidney transcriptome of brown trout during active *T. bryosalmonae* sporogenesis [25]. The aim of this experiment was to gain insight about these TLRs and to investigate their expression patterns from an early phase (2 wpe) to late phase (17 wpe) of PKD in brown trout. TLR1 and TLR19 had a single copy of the gene in the brown trout genome, whereas we found multiples copies of TLR13-like genes localized at different chromosomes of brown trout. Therefore, we evaluated TLR1, TLR19, and four TLR13-like genes i.e., TLR13-like Chr1, TLR13-like Chr2, TLR13-like Chr6, and TLR13-like Chr27 for comparison.

In the present study, the gene nomenclature of two TLRs originally designated as TLR6 and TLR12 in the brown trout genome assembly was re-annotated as TLR1 and TLR19 based on the model animal zebrafish. Moreover, previous studies suggest that TLR6 and TLR12 are absent in teleost [36,37,38]. Similar inconsistencies in the gene nomenclature has been reported in the orthologous connexin genes of teleost [43]. Incorrect gene nomenclature and the mis-annotation of genes in the genomic resources may occur due to the automated annotation process during genome assembly [44,45,46]. Likewise, structural and functional mis-annotations have been reported from the human malaria parasite (*Plasmodium vivax*) genome [47], fungal genomes [48], and plant genomes [49,50]. Therefore, it is important to re-annotate or curate the genes mined from the genomic resources available in the public databases. A local comparative synteny analysis provides a framework of conserved homologous gene order along with their transcriptional direction. Synteny analysis is one of the effective methods to re-annotate and to rectify the inconsistencies in the nomenclature of orthologous genes across species [39]. Furthermore, phylogenetic analysis can support the results of synteny analysis by looking into the evolutionary relationship between the orthologous genes [51]. In the present study, re-annotation of TLR1 and TLR19 is supported by both synteny and phylogenetic analysis.

The first report of TLR1 in teleost was from pufferfish (*Fugu rubripes*) [52], and it is characterized in many fish species including rainbow trout [53]. In higher vertebrates, TLR1 is known to detect dipalmitoylated and triacylated lipoproteins derived from *Mycoplasma pneumonia* [54,55]. In humans, TLR1 and TLR6 are localized in chromosome 4 in proximal tandem as a result of tandem gene duplication [56,57]. However, teleost do not have TLR6 [36,37,38], and furthermore, the present study also confirms the absence of TLR6 in brown trout. Moreover, the phylogenetic analysis clustered TLR1 amino acid sequences from various vertebrates as a separate monophyletic clade (Figure 5). Brown trout TLR1 had five LRR domains, one LRR-CT domain, and no TM region (Figure 3). An analogous domain architecture was observed in the TLR1 of other fish species, for example as rainbow trout [53], pufferfish [52], zebrafish [58], orange-spotted grouper (*Epinephelus coioides*) [59], and large yellow croaker (*Pseudosciaena crocea*) [60]. A signal peptide was observed on the N-terminal region of brown trout TLR1 and it might be flagged toward the secretion pathway [61]. Similarly, a signal peptide was observed in the orthologous TLR1 of other vertebrates including human, mouse, chicken (*Gallus gallus*), zebrafish, grass carp (*Ctenopharyngodon idella*), channel catfish, barred knifejaw (*Oplegnathus fasciatus*), rainbow trout, Japanese flounder (*Paralichthys olivaceus*), orange-spotted grouper, and large yellow croaker [53,59,60]. We observed a significant up-regulation of TLR1 in the kidney of brown trout from 6–12 wpe (Figure 6A). Similarly, TLR1 was up-regulated in channel catfish [33] and naked carp (*Gymnocypris przewalskii*) [62] in response to *Ichthyophthirius multifiliis* and in orange-spotted grouper during *Cryptocaryon irritans* infection [63]. In humans, genetic variation in TLR1 gene is known to influence the parasite burden during malaria caused by *Plasmodium falciparum* [64]. The regulation of TLR1 in fish may have a protective role by inducing host immune system against the evading parasitic pathogen.

The TLR19 of fish is well conserved and is closely related to the TLR11, TLR12, and TLR20 of other vertebrates [65]. The first characterization of TLR19 among teleost was from zebrafish [66]. In addition, TL19 has been reported from Atlantic salmon [34], channel catfish [38,67], grass carp [68,69], and blunt snout bream (*Megalobrama amblycephala*) [70]. TLR19 is known for the antiviral immunity in grass carp by detecting dsRNA analogs and promoting the expression of IFN and NF-κB [68]. In the present study, TLR19 was up-regulated in the kidney of brown trout from 6–12 wpe (Figure 6B). In the study of Lee et al. [34], TLR19 was also significantly up-regulated in the kidney grade 1-2, grade 2, and grade 3 of PKD affected rainbow trout. It is evident that TLR19 plays an important role against *T. bryosalmonae* in both brown trout and rainbow trout. Similar tissue-specific up-regulation of TLR19 was also noticed in the skin and gill of channel catfish during the infection of *I. multifiliis* [33].

TLR13 belongs to TLR11 subfamily, and it has been reported from fishes such as miiuy croaker (*Miichthys miiuy*) [71], orange-spotted grouper [72], Atlantic salmon, and coho salmon [73]. Conserved bacterial 23S ribosomal RNA sequences could be recognized by mouse TLR13 and further activates the MyD88-dependent signaling cascade [74,75]. However, only fewer experiments were done in fish to understand the exact function of TLR13. In the present study, we examined the expression pattern of TLR13-like genes in the posterior kidney of brown trout in response to *T. bryosalmonae*. TLR13-like Chr1 and TLR13-like Chr6 have a signal peptide in the N-terminal region and can enter the secretion pathway. Furthermore, among TLR13-like genes, TLR13-like chr1 showed a strong differential expression (4–12 wpe) compared to other TLR13-like genes in the brown trout (Figure 7). Likewise, TLR13 was up-regulated in the skin of Atlantic salmon and coho salmon infected with sea louse (*Caligus rogercresseyi*) [73]. Experiments in the leukocytes of miiuy croaker suggest that poly (I:C) and *Vibrio anguillarum* can promote TLR13 expression [71]. Taken together, it is possible that TLR13 plays an important role against pathogen invasion in fish.

All the evaluated TLRs showed a significant up-regulation in the posterior kidney of brown trout in response to *T. bryosalmonae* from 6–10 wpe (Figure 6 and Figure 7). Interestingly, typical clinical signs of PKD such as renal hyperplasia, splenomegaly, and pale liver were also observed in the kidney of brown trout from 6 to 12 wpe. The pathogen invasion activates TLRs that can mediate a cytokine response that induces an inflammatory reaction in the host tissues [76,77]. Similarly, differential expression of various cytokines and their receptors were observed in the posterior kidney of brown trout during active *T. bryosalmonae* proliferation [25]. In the present study, we could neither observe a clinical sign of the disease nor a gene regulation of all examined TLRs at the early (2 wpe) and late (17 wpe) phases of parasite infection in brown trout. During the early phase (2 wpe) of *T. bryosalmonae* infection in brown trout, the parasite might manage to evade from the host immune system and clandestinely reach its target organ kidney. However, the non-regulation of TLRs at the late phase (17 wpe) of infection may favor asymptomatic brown trout-*T. bryosalmonae* coexistence. However, further studies are needed to evaluate this hypothesis.

## 4. Materials and Methods

### 4.1. Brown Trout Experiment

The present study is a continuation of our previous research work, and the details of the experiment are described in Sudhagar et al. [25]. Briefly, specific pathogen-free brown trout were exposed to *T. bryosalmonae*. Then, the posterior kidney of fish was sampled at 2, 4, 6, 8, 10, 12, and 17 wpe. The sampled tissues were fixed in 10% neutral-buffered formalin (NBF) for histology and RNA*later* (Sigma, Steinheim, Germany) for molecular studies. During sampling, the fishes were observed for typical clinical signs of PKD such as renal hyperplasia, splenomegaly, and pale liver. This experiment was conducted in accordance with the relevant guidelines and regulations §26 of the Austrian Law for Animal Experiments, Tierversuchsgesetz 2012. The institutional ethics committee of the University of Veterinary Medicine, Vienna, Austria and the national authority approved this experiment under the permission number BMWFW-68.205/0181-WF/V/3b/2017.

### 4.2. Histology and Immunohistochemistry

NBF-fixed tissue samples were processed for histology, and tissue sections were mounted on glass slides. The slides were then deparaffinized and either stained with hematoxylin and eosin (H&E) for histopathological observation or processed for IHC to visualize *T. bryosalmonae*. For IHC, the tissue sections were treated with P01 - *T. bryosalmonae* specific monoclonal antibody (Aquatic Diagnostics, Stirling, UK) according to the manufacturer’s instruction. Subsequently, VECTASTAIN^®^ ABC HRP Kit (Vector laboratories, Burlingame, CA, USA) was used for the visualization of antigen–antibody reaction (i.e., *T. bryosalmonae*) on the tissue sections. The sections were counterstained with hematoxylin, mounted, and examined for parasite stages under a microscope.

### 4.3. Identification and Sequence Analysis of TLRs

In our earlier experiment, three TLRs were differentially up-regulated in the transcriptome of the kidney of brown trout during PKD pathogenesis [25]. The nucleic acid sequences of these TLRs were used as queries against the recently released brown trout genome (NCBI *Salmo trutta* annotation release 100, accession number: GCF_901001165.1) using the BLASTX and BLASTN. Then, the obtained protein accession numbers and the nucleic acid alignment results were mapped on the NCBI genome data viewer for *Salmo trutta* to mine out the details of corresponding TLR mRNA sequence, amino acid sequence, orientation, and chromosome location. Among them, two mined sequences XP_029587338.1 and XP_029602002.1 were originally named as TLR6 and TLR12, respectively and each with a single copy in the genome. However, Ensembl track (*Salmo trutta* release 99) in the genome data viewer tool demonstrated these two TLRs as TLR1 (ENSSTUG00000033178) and TLR19 (ENSSTUG00000029468), respectively. In support of this, the absence of TLR6 and TLR12 has already been reported in teleost fish [36,37,38]. The third mined TLR sequence was identified as the TLR13-like gene located on chromosome 1 (TLR13-like Chr1); however, multiple copies of TLR13-like genes were found in the genome. Additionally, for comparison, TLR13-like genes located in chromosome 2 (TLR13-like Chr2), chromosome 6 (TLR13-like Chr6), and chromosome 27 (TLR13-like Chr27) were also analyzed in the present study. The ProtComp 9.0 tool (http://www.softberry.com) and ProtParam tool (https://web.expasy.org/protparam/) were used for subcellular localization and MW and pI predication of TLRs respectively. The SMART tool was used to predict the conserved domain architecture of TLRs along with option selected to detect signal peptide regions based on the SignaIP4.0 server.

### 4.4. Synteny Analysis

The genomic neighborhood arrangements of brown trout TLR1 and TLR19 were compared with the orthologous TLRs of rainbow trout and zebrafish using local synteny analysis. Synteny analysis further confirmed the gene nomenclature of brown trout TLR1 and TLR19. The amino acid sequences of brown trout TLRs were provided as queries in the Genomicus database to visualize the synteny block arrangement of the corresponding orthologous TLRs in the model organism zebrafish [78]. The chromosomal localization, transcriptional orientation, and neighborhood genes of the TLRs of brown trout and rainbow trout were retrieved using the genome data viewer of NCBI and comparative genomics alignment viewer of ENSEMBL.

### 4.5. Phylogenetic Analysis

The sequence alignment and phylogenetic tree construction of TLRs were performed in MEGA X [79]. Amino acid sequences of TLRs of brown trout and other vertebrate representatives (Supplementary material Table S2) were aligned using the MUSCLE program with default parameters. The best-fitting models of sequence evolution were determined with ‘Find Best Protein Models’ supplemented in MEGA X with complete deletion of gaps. The Jones–Taylor–Thornton model along with discrete gamma distributed with invariant sites (JTT+G+I) was determined as the best model based on the maximum log-likelihood value (lnL: –11422.216) and lowest Bayesian information criterion score (24151.057). The maximum likelihood method was used to construct the phylogenetic tree using the JTT+G+I model. The initial tree used for the heuristic search was obtained by applying the default neighbor-joining and BioNJ algorithms. All positions containing gaps and missing data were eliminated, and the bootstrapping value was set as 1000 replications. The final tree was generated with interactive tree of life (iTOL, http://itol.embl.de/).

### 4.6. RNA Extraction and Quantitative Real Time PCR

To understand the biological function of TLRs during PKD pathogenesis in brown trout, their expression pattern was visualized in the posterior kidney of brown trout at 2, 4, 6, 8, 10, 12 and 17 wpe in response to *T. bryosalmonae*. Total RNA was extracted from individual posterior kidney of exposed and control brown trout (*n* = 9) using RNeasy Mini Kit (Qiagen, Hilden, Germany) with an on-column DNase I digestion step (Qiagen, Hilden, Germany). The quality and quantity of extracted total RNA were assessed using NanoDrop 2000 spectrophotometer (Thermo Fisher Scientific, Vienna, Austria), and by RNA ScreenTape assay using 4200 TapeStation (Agilent, Santa Clara, CA, USA). cDNA synthesize was done using the iScript cDNA synthesis kit (Bio-Rad, Hercules, USA) with an input of one µg total RNA.

The NCBI Primer-BLAST online tool was used to design gene-specific primers according to the mRNA sequence data of all the six TLRs identified from the genome. All the TLR gene-specific primers were designed targeting genes with no preference on the exon junction span. The primers were optimized for annealing temperature, and the single PCR products were sequenced to substantiate the specificity and sensitivity of primers. Furthermore, the primers were checked for their efficiency before proceeding for expression analysis.

Individual samples of exposed and control kidneys at different time points were subjected to qRT-PCR with technical replicates using selected gene primers. No-template control and no-reverse transcriptase control were used to assess the performance of the qRT-PCR assay. The final reaction volume for qRT-PCR was 10 μL, which includes 5 μL of 2X SsoAdvanced™ Universal SYBR Green Supermix (Bio-Rad), 0.5 μL of each primer (10 pmol/μL), 1 μL DEPC-treated sterile distilled water (Carl Roth, Karlsruhe, Germany), and 3 μL of 1:20-fold diluted cDNA. After initial cDNA denaturation at 95 °C for 5 min, 37 cycles of denaturation (95 °C for 30 s), annealing (61 °C for 30 s) and extension (72 °C for 30 s) were performed in a CFX96 Touch Real-Time PCR detection system (Bio-Rad, München, Germany). Melting curve analysis was performed at the end of all gene expression cycling protocols under the conditions of 61 °C for 30 s to 95 °C with an increment of 0.5 °C for 10 s. The test samples were normalized using elongation factor-alpha and beta-actin as the reference genes [80]. The relative gene expression between the exposed and control group was determined using the 2^−ΔΔCt^ method. The details of the primers designed in the present study are provided in Table 2.

### 4.7. Statistical Analysis

The relative fold change values were tested for tank effect using nested ANOVA (tanks nested under groups), and no significant difference was observed between the replicates. The statistical difference between exposed and control groups at each time point was calculated using the two-tailed Student’s t-test. The statistical analysis was performed in R statistical software version 3.5.1 and *p*-value < 0.05 was considered significant [81]. Heatmap representation of expression profile of TLRs was generated using heatmap.2 function of gplots package and hierarchical clustering based on Euclidean distance and complete linkage.

## 5. Conclusions

In conclusion, we provided information about the selected TLRs in brown trout using data-mining, synteny and phylogenetic analysis. Protein domain, phylogenetic, and syntenic analysis showed that TLR1, TLR19, and TLR13-like genes of brown trout are conserved across fish species. This is the first study on brown trout to evaluate and provide fundamental information regarding TLR expression patterns in response to myxozoan infection. The present study showed that the expression levels of TLRs were highly up-regulated in the kidney of brown trout during the development of *T. bryosalmonae*. All the evaluated TLRs had their peak expression from 6 to 10 wpe, and our results suggest that TLRs might play a pivotal role in the immune response of brown trout during PKD pathogenesis. However, further studies are needed for the complete characterization of different TLR paralogs in brown trout and evaluate their response during *T. bryosalmonae* infection.

## Figures and Tables

**Table 1:** Characteristics of Toll-like receptors (TLRs) of brown trout used in the present study. The chromosome number, location, transcriptional direction (F–forward and R–reverse), NCBI accession numbers, length of mRNA, and the amino acid sequences of each TLR are provided. The details of the predicted isoelectric point (pI), molecular weight (MW), and subcellular location are also shown.

TLRs	Chromosome	Location	Orientation	mRNA Accession Number	mRNA Length	Protein Accession Number	Amino Acid Length	pI	MW (kDa)	Subcellular Location
TLR1	3	32933053–32935476	R	XM_029731478.1* (TLR6)	3149	XP_029587338.1* (TLR6)	807	6.23	90.8	Plasma membrane
TLR19	3	70531574–70546575	F	XM_029746142.1* (TLR12)	5815	XP_029602002.1* (TLR12)	972	6.15	109.9	Plasma membrane
TLR13-like Chr1	1	76708863–76711946	F	XM_029775870.1	4057	XP_029631730.1	1027	6.01	117.4	Membrane bound endoplasmic reticulum
TLR13-like Chr2	2	17203261–17205151	R	XM_029691145.1	1422	XP_029547005.1	322	6.73	38.4	Plasma membrane
TLR13-like Chr6	6	49945705–49948596	R	XM_029756725.1	3799	XP_029612585.1	963	7.32	109.8	Membrane bound mitochondria
TLR13-like Chr27	27	28493214–28496051	F	XM_029717624.1	3451	XP_029573484.1	945	6.42	108.4	Membrane bound vacuoles

* Unlike higher vertebrates, bony fish do not have TLR6 and TLR12 [36,37,38]. Based on our local synteny analysis, the accession numbers of the originally described TLR6 and TLR12 were re-annotated as TLR1 and TLR19, respectively.

**Table 2:** List of quantitative qRT-PCR primers designed in this study.

Gene	Forward (5′-3′)	Amplicon	Accession Number
TLR1	Forward: TCGAAATCTGATCGCAGACG Reverse: CAAGGCGTTTATGGTGCTTG	187	XM_029731478.1
TLR19	Forward: CTAACCCATTCAAAATCCTGTACCC Reverse: TTGGACATCTTTCACAAATGCTAT	150	XM_029746142.1
TLR13 Chr1	Forward: TGCTGCTCCTTCGATGC Reverse: CACCCTGCAGTTGAATTGTATC	175	XM_029775870.1
TLR13 Chr2	Forward: GGAAGCTATGATGTAATTGTCTGC Reverse: CGCTAGTGCATAAGCTGGAA	158	XM_029691145.1
TLR13 Chr6	Forward: CTAGGCTGCTCAGATTTCGC Reverse: AGGCATCATTCAAGGTCAGG	157	XM_029756725.1
TLR13 Chr27	Forward: CTGGGAAGTGAACCCTGAG Reverse: GTCCGTTAAATGTAAAGTCCGAAA	197	XM_029717624.1

## Supplementary Materials

Supplementary materials can be found at https://www.mdpi.com/1422-0067/21/11/3755/s1.

## Abbreviations

H&E	Hematoxylin and eosin
IHC	Immunohistochemistry
IL-1	Interleukin-1
JTT+G+I	Jones–Taylor–Thornton model along with discrete gamma distributed with invariant sites
lnL	Log-likelihood
LRR-CT	LRR C-terminal
LRR TYP	Typical LRR
LRRs	Leucine-rich repeats
MW	Molecular weight
NBF	Neutral-buffered formalin
PAMPS	Pathogen-associated molecular patterns
pI	Isoelectric point
PKD	Proliferative kidney disease
PRRs	Pattern recognition receptors
qRT-PCR	Quantitative real time PCR
TIR	Toll/interleukin-1 receptor
TLRs	Toll-like receptors
TM	Transmembrane
wpe	Weeks post-exposure

## References

[1] Canning E.U., Curry A., Feist S.W., Longshaw M., Okamura B. (1999). *Tetracapsula bryosalmonae* n.sp. for PKX organism, the cause of PKD in salmonid fish. Bull. Eur. Assoc. Pathol..

[2] Henderson M., Okamura B. (2004). The phylogeography of salmonid proliferative kidney disease in Europe and North America. Proc. R. Soc. B Biol. Sci..

[3] Sudhagar A., Kumar G., El-Matbouli M. (2020). The malacosporean myxozoan parasite *Tetracapsuloides bryosalmonae*: A threat to wild salmonids. Pathogens.

[4] Wahli T., Knuesel R., Bernet D., Segner H., Pugovkin D., Burkhardt-Holm P., Escher M., Schmidt-Posthaus H. (2002). Proliferative kidney disease in Switzerland: Current state of knowledge. J. Fish Dis..

[5] Waldner K., Bechter T., Auer S., Borgwardt F., El-Matbouli M., Unfer G. (2019). A brown trout (*Salmo trutta*) population faces devastating consequences due to proliferative kidney disease and temperature increase: A case study from Austria. Ecol. Freshw. Fish.

[6] Yellowstone River Fish Kill Fact Sheet-Updated. http://fwp.mt.gov/news/newsReleases/headlines/nr_4278.html.

[7] Sage J.L. (2016). Economic Contributions of the Yellowstone River to Park County, Montana.

[8] Morris D.J., Adams A. (2006). Transmission of *Tetracapsuloides bryosalmonae* (Myxozoa: Malacosporea), the causative organism of salmonid proliferative kidney disease, to the freshwater bryozoan *Fredericella sultana*. Parasitology.

[9] Grabner D.S., El-Matbouli M. (2008). Transmission of *Tetracapsuloides bryosalmonae* (Myxozoa: Malacosporea) to *Fredericella sultana* (Bryozoa: Phylactolaemata) by various fish species. Dis. Aquat. Org..

[10] Grabner D.S., El-Matbouli M. (2010). *Tetracapsuloides bryosalmonae* (Myxozoa: Malacosporea) portal of entry into the fish host. Dis. Aquat. Org..

[11] Morris D.J., Adams A. (2008). Sporogony of *Tetracapsuloides bryosalmonae* in the brown trout *Salmo trutta* and the role of the tertiary cell during the vertebrate phase of myxozoan life cycles. Parasitology.

[12] Clifton-Hadley R., Feist S. (1989). Proliferative kidney disease in brown trout *Salmo trutta* further evidence of a myxosporean aetiology. Dis. Aquat. Organ..

[13] Chilmonczyk S., Monge D., De Kinkelin P. (2002). Proliferative kidney disease: Cellular aspects of the rainbow trout, *Oncorhynchus mykiss* (Walbaum), response to parasitic infection. J. Fish Dis..

[14] Schmidt-Posthaus H., Bettge K., Forster U., Segner H., Wahli T. (2012). Kidney pathology and parasite intensity in rainbow trout *Oncorhynchus mykiss* surviving proliferative kidney disease: Time course and influence of temperature. Dis. Aquat. Org..

[15] Kumar G., Abd-Elfattah A., Saleh M., El-Matbouli M. (2013). Fate of *Tetracapsuloides bryosalmonae* (Myxozoa) after infection of brown trout *Salmo trutta* and rainbow trout *Oncorhynchus mykiss*. Dis. Aquat. Organ..

[16] Soliman H., Kumar G., El-Matbouli M. (2018). *Tetracapsuloides bryosalmonae* persists in brown trout *Salmo trutta* for five years post exposure. Dis. Aquat. Org..

[17] Bailey C., Segner H., Casanova-Nakayama A., Wahli T. (2017). Who needs the hotspot? The effect of temperature on the fish host immune response to *Tetracapsuloides bryosalmonae* the causative agent of proliferative kidney disease. Fish Shellfish Immunol..

[18] Holland J.W., Gould C.R.W., Jones C.S., Noble L.R., Secombes C.J. (2003). The expression of immune-regulatory genes in rainbow trout, *Oncorhynchus mykiss*, during a natural outbreak of proliferative kidney disease (PKD). Parasitology.

[19] Gorgoglione B., Wang T., Secombes C.J., Holland J.W. (2013). Immune gene expression profiling of proliferative kidney disease in rainbow trout *Oncorhynchus mykiss* reveals a dominance of anti-inflammatory, antibody and Th cell-like activities. Vet. Res..

[20] Bailey C., Segner H., Wahli T. (2017). What goes around comes around: An investigation of resistance to proliferative kidney disease in rainbow trout *Oncorhynchus mykiss* (Walbaum) following experimental re-exposure. J. Fish Dis..

[21] Abos B., Estensoro I., Perdiguero P., Faber M., Hu Y., Rosales P.D., Granja A.G., Secombes C.J., Holland J.W., Tafalla C. (2018). Dysregulation of B cell activity during proliferative kidney disease in rainbow trout. Front. Immunol..

[22] Kumar G., Abd-Elfattah A., El-Matbouli M. (2015). Identification of differentially expressed genes of brown trout (*Salmo trutta*) and rainbow trout (*Oncorhynchus mykiss*) in response to *Tetracapsuloides bryosalmonae* (Myxozoa). Parasitol. Res..

[23] Kotob M.H., Kumar G., Saleh M., Gorgoglione B., Abdelzaher M., El-Matbouli M. (2018). Differential modulation of host immune genes in the kidney and cranium of the rainbow trout (*Oncorhynchus mykiss*) in response to *Tetracapsuloides bryosalmonae* and *Myxobolus cerebralis* co-infections. Parasites Vectors.

[24] Bailey C., Strepparava N., Wahli T., Segner H. (2019). Exploring the immune response, tolerance and resistance in proliferative kidney disease of salmonids. Dev. Comp. Immunol..

[25] Sudhagar A., Ertl R., Kumar G., El-Matbouli M. (2019). Transcriptome profiling of posterior kidney of brown trout, *Salmo trutta*, during proliferative kidney disease. Parasit. Vectors.

[26] Kumar G., Gotesman M., El-Matbouli M. (2015). Interaction of *Tetracapsuloides bryosalmonae*, the causative agent of proliferative kidney disease, with host proteins in the kidney of *Salmo trutta*. Parasitol. Res..

[27] Kumar G., Sarker S., Menanteau-Ledouble S., El-Matbouli M. (2015). *Tetracapsuloides bryosalmonae* infection affects the expression of genes involved in cellular signal transduction and iron metabolism in the kidney of the brown trout *Salmo trutta*. Parasitol. Res..

[28] Wallet S.M., Puri V., Gibson F.C. (2018). Linkage of infection to adverse systemic complications: Periodontal disease, toll-like receptors, and other pattern recognition systems. Vaccines.

[29] Takeda K., Kaisho T., Akira S. (2003). Toll—Like receptors. Annu. Rev. Immunol..

[30] Aguirre-García M.M., Rojas-Bernabé A., Gómez-García A.P., Escalona-Montaño A.R., Rezaei N. (2020). TLR-mediated host immune response to parasitic infectious diseases. Toll-Like Receptors.

[31] Rajasekaran S., Anuradha R., Bethunaickan R. (2017). TLR specific immune responses against helminth infections. J. Parasitol. Res..

[32] Tu X., Liu L., Qi X., Chen W., Wang G., Ling F. (2016). Characterization of Toll-like receptor gene expression in goldfish (*Carassius auratus*) during *Dactylogyrus intermedius* infection. Dev. Comp. Immunol..

[33] Zhao F., Li Y.W., Pan H.J., Shi C.B., Luo X.C., Li A.X., Wu S.Q. (2013). Expression profiles of toll-like receptors in channel catfish (*Ictalurus punctatus*) after infection with *Ichthyophthirius multifiliis*. Fish Shellfish Immunol..

[34] Lee P.T., Zou J., Holland J.W., Martin S.A.M., Collet B., Kanellos T., Secombes C.J. (2014). Identification and characterisation of TLR18-21 genes in Atlantic salmon (*Salmo salar*). Fish Shellfish Immunol..

[35] Moreira G.S.A., Shoemaker C.A., Zhang D., Xu D.H. (2017). Expression of immune genes in skin of channel catfish immunized with live theronts of *Ichthyophthirius multifiliis*. Parasite Immunol..

[36] Nie L., Cai S.Y., Shao J.Z., Chen J. (2018). Toll-like receptors, associated biological roles, and signaling networks in non-mammals. Front. Immunol..

[37] Fan H., Wang L., Wen H., Wang K., Qi X., Li J., He F., Li Y. (2019). Genome-wide identification and characterization of toll-like receptor genes in spotted sea bass (*Lateolabrax maculatus*) and their involvement in the host immune response to *Vibrio harveyi* infection. Fish Shellfish Immunol..

[38] Quiniou S.M.A., Boudinot P., Bengtén E. (2013). Comprehensive survey and genomic characterization of Toll-like receptors (TLRs) in channel catfish, *Ictalurus punctatus*: Identification of novel fish TLRs. Immunogenetics.

[39] Jun J., Mandoiu I.I., Nelson C.E. (2009). Identification of mammalian orthologs using local synteny. BMC Genom..

[40] Sudhagar A., Kumar G., El-Matbouli M. (2018). Transcriptome analysis based on RNA-Seq in understanding pathogenic mechanisms of diseases and the immune system of fish: A comprehensive review. Int. J. Mol. Sci..

[41] Eggestøl H.O., Lunde H.S., Rønneseth A., Fredman D., Petersen K., Mishra C.K., Furmanek T., Colquhoun D.J., Wergeland H.I., Haugland G.T. (2018). Transcriptome-wide mapping of signaling pathways and early immune responses in lumpfish leukocytes upon in vitro bacterial exposure. Sci. Rep..

[42] Kumar G., Ertl R., Bartholomew J.L., El-Matbouli M. (2020). First transcriptome analysis of bryozoan *Fredericella sultana*, the primary host of myxozoan parasite *Tetracapsuloides bryosalmonae*. PeerJ.

[43] Mikalsen S.O., Tausen M., Í Kongsstovu S. (2020). Phylogeny of teleost connexins reveals highly inconsistent intra- and interspecies use of nomenclature and misassemblies in recent teleost chromosome assemblies. BMC Genom..

[44] Nobre T., Campos M.D., Lucic-Mercy E., Arnholdt-Schmitt B. (2016). Misannotation awareness: A tale of two gene-groups. Front. Plant Sci..

[45] Schnoes A.M., Brown S.D., Dodevski I., Babbitt P.C. (2009). Annotation error in public databases: Misannotation of molecular function in enzyme superfamilies. PLoS Comput. Biol..

[46] Zallot R., Harrison K.J., Kolaczkowski B., De Crécy-Lagard V. (2016). Functional annotations of paralogs: A blessing and a curse. Life.

[47] Arévalo-Pinzón G., Curtidor H., Abril J., Patarroyo M.A. (2013). Annotation and characterization of the *Plasmodium vivax* rhoptry neck protein 4 (PvRON4). Malar. J..

[48] Stergiopoulos I., Kourmpetis Y.A.I., Slot J.C., Bakker F.T., De Wit P.J.G.M., Rokas A. (2012). In silico characterization and molecular evolutionary analysis of a novel superfamily of fungal effector proteins. Mol. Biol. Evol..

[49] Sun X., Wang G.L. (2011). Genome-wide identification, characterization and phylogenetic analysis of the rice LRR-kinases. PLoS ONE.

[50] Li Y., Wei W., Feng J., Luo H., Pi M., Liu Z., Kang C. (2018). Genome re-annotation of the wild strawberry *Fragaria vesca* using extensive Illumina-and SMRT-based RNA-seq datasets. DNA Res..

[51] Hausken K., Levavi-Sivan B. (2019). Synteny and phylogenetic analysis of paralogous thyrostimulin beta subunits (GpB5) in vertebrates. PLoS ONE.

[52] Oshiumi H., Tsujita T., Shida K., Matsumoto M., Ikeo K., Seya T. (2003). Prediction of the prototype of the human Toll-like receptor gene family from the pufferfish, *Fugu rubripes*, genome. Immunogenetics.

[53] Palti Y., Rodriguez M.F., Gahr S.A., Purcell M.K., Rexroad C.E., Wiens G.D. (2010). Identification, characterization and genetic mapping of TLR1 loci in rainbow trout (*Oncorhynchus mykiss*). Fish Shellfish Immunol..

[54] Shimizu T., Kida Y., Kuwano K. (2007). Triacylated lipoproteins derived from *Mycoplasma pneumoniae* activate nuclear factor-κB through toll-like receptors 1 and 2. Immunology.

[55] Shimizu T., Kida Y., Kuwano K. (2005). A Dipalmitoylated lipoprotein from *Mycoplasma pneumoniae* activates NF-κB through TLR1, TLR2, and TLR6. J. Immunol..

[56] Rock F.L., Hardiman G., Timans J.C., Kastelein R.A., Bazan J.F. (1998). A family of human receptors structurally related to Drosophila Toll. Proc. Natl. Acad. Sci. USA.

[57] Takeuchi O., Kawai T., Sanjo H., Copeland N.G., Gilbert D.J., Jenkins N.A., Takeda K., Akira S. (1999). TLR6: A novel member of an expanding Toll-like receptor family. Gene.

[58] Jault C., Pichon L., Chluba J. (2004). Toll-like receptor gene family and TIR-domain adapters in *Danio rerio*. Mol. Immunol..

[59] Wei Y.C., Pan T.S., Chang M.X., Huang B., Xu Z., Luo T.R., Nie P. (2011). Cloning and expression of Toll-like receptors 1 and 2 from a teleost fish, the orange-spotted grouper *Epinephelus coioides*. Vet. Immunol. Immunopathol..

[60] Wang K., Mu Y., Qian T., Ao J., Chen X. (2013). Molecular characterization and expression analysis of Toll-like receptor 1 from large yellow croaker (*Pseudosciaena crocea*). Fish Shellfish Immunol..

[61] Owji H., Nezafat N., Negahdaripour M., Hajiebrahimi A., Ghasemi Y. (2018). A comprehensive review of signal peptides: Structure, roles, and applications. Eur. J. Cell Biol..

[62] Tong C., Lin Y., Zhang C., Shi J., Qi H., Zhao K. (2015). Transcriptome-wide identification, molecular evolution and expression analysis of Toll-like receptor family in a Tibet fish, *Gymnocypris przewalskii*. Fish Shellfish Immunol..

[63] Li Y.W., Xu D.D., Li X., Mo Z.Q., Luo X.C., Li A.X., Dan X.M. (2016). Identification and characterization of three TLR1 subfamily members from the orange-spotted grouper, *Epinephelus coioides*. Dev. Comp. Immunol..

[64] Hahn W.O., Harju-Baker S., Erdman L.K., Krudsood S., Kain K.C., Wurfel M.M., Liles W.C. (2016). A common TLR1 polymorphism is associated with higher parasitaemia in a Southeast Asian population with *Plasmodium falciparum* malaria. Malar. J..

[65] Wang J., Zhang Z., Fu H., Zhang S., Liu J., Chang F., Li F., Zhao J., Yin D. (2015). Structural and evolutionary characteristics of fish-specific TLR19. Fish Shellfish Immunol..

[66] Meijer A.H., Gabby Krens S.F., Medina Rodriguez I.A., He S., Bitter W., Snaar-Jagalska B.E., Spaink H.P. (2004). Expression analysis of the Toll-like receptor and TIR domain adaptor families of zebrafish. Mol. Immunol..

[67] Zhang J., Liu S., Rajendran K.V., Sun L., Zhang Y., Sun F., Kucuktas H., Liu H., Liu Z. (2013). Pathogen recognition receptors in channel catfish: III Phylogeny and expression analysis of Toll-like receptors. Dev. Comp. Immunol..

[68] Ji J., Rao Y., Wan Q., Liao Z., Su J. (2018). Teleost-specific TLR19 localizes to endosome, recognizes dsRNA, recruits TRIF, triggers both IFN and NF-κB pathways, and protects cells from grass carp reovirus infection. J. Immunol..

[69] Liao Z., Wan Q., Su H., Wu C., Su J. (2017). Pattern recognition receptors in grass carp *Ctenopharyngodon idella*: I. Organization and expression analysis of TLRs and RLRs. Dev. Comp. Immunol..

[70] Lai R.F., Jakovlić I., Liu H., Zhan F.B., Wei J., Wang W.M. (2017). Molecular characterization and immunological response analysis of toll-like receptors from the blunt snout bream (*Megalobrama amblycephala*). Dev. Comp. Immunol..

[71] Wang Y., Bi X., Chu Q., Xu T. (2016). Discovery of toll-like receptor 13 exists in the teleost fish: Miiuy croaker (Perciformes, Sciaenidae). Dev. Comp. Immunol..

[72] Liang Y., Ding X., Yu X., Wang Y., Zhou Y., He J., Shi Y., Zhang Y., Lin H., Lu D. (2018). Identification and functional characterization of Toll-like receptor 13 from orange-spotted grouper (*Epinephelus coioides*). Fish Shellfish Immunol..

[73] Valenzuela-Muñoz V., Boltaña S., Gallardo-Escárate C. (2016). Comparative immunity of *Salmo salar* and *Oncorhynchus kisutch* during infestation with the sea louse *Caligus rogercresseyi*: An enrichment transcriptome analysis. Fish Shellfish Immunol..

[74] Oldenburg M., Krüger A., Ferstl R., Kaufmann A., Nees G., Sigmund A., Bathke B., Lauterbach H., Suter M., Dreher S. (2012). TLR13 recognizes bacterial 23S rRNA devoid of erythromycin resistance-forming modification. Science.

[75] Hochrein H., Kirschning C.J. (2013). Bacteria evade immune recognition via TLR13 and binding of their 23S rRNA by MLS antibiotics by the same mechanisms. Oncoimmunology.

[76] Grassin-Delyle S., Abrial C., Salvator H., Brollo M., Naline E., Devillier P. (2020). The role of Toll-like receptors in the production of cytokines by human lung macrophages. J. Innate Immun..

[77] Strieter R.M., Belperio J.A., Keane M.P. (2003). Host innate defenses in the lung: The role of cytokines. Curr. Opin. Infect. Dis..

[78] Muffato M., Louis A., Poisnel C.E., Crollius H.R. (2010). Genomicus: A database and a browser to study gene synteny in modern and ancestral genomes. Bioinformatics.

[79] Kumar S., Stecher G., Li M., Knyaz C., Tamura K. (2018). MEGA X: Molecular evolutionary genetics analysis across computing platforms. Mol. Biol. Evol..

[80] Kumar G., Abd-Elfattah A., El-Matbouli M. (2014). Differential modulation of host genes in the kidney of brown trout *Salmo trutta* during sporogenesis of *Tetracapsuloides bryosalmonae* (Myxozoa). Vet. Res..

[81] R Core Team R: A Language and Environment for Statistical Computing; R Foundation for Statistical Computing. https://www.r-project.org/.

