# Supplementary material for: Identification and Expression Profiling of Toll-Like Receptors of Brown Trout (Salmo trutta) during Proliferative Kidney Disease
Source: Int J Mol Sci. 2020 May 26;21(11):3755. doi: 10.3390/ijms21113755 (PMC7312180; doi:10.3390/ijms21113755)
Supplement: Supplementary file 1 [file ijms-21-03755-s001.pdf]

Supplementary Table S1: Abbreviation of gene symbols used in synteny analysis

Sl	Gene symbol	Abbreviation
1	ADRA2DA	Alpha-2Da adrenergic receptor
2	CGNA	Cingulin A
3	CHRNA9	Neuronal acetylcholine receptor subunit alpha-9
4	CHRNB2	Neuronal acetylcholine receptor subunit beta-2
5	FLAD1	FAD synthase
6	FRMPD1	FERM and PDZ domain-containing protein 1
7	INTS10	Integrator complex subunit 10
8	KLB	Beta-klotho
9	LENEP	Lens epithelial cell protein LEP503
10	PBXIP1B	Pre-B-cell leukemia transcription factor-interacting protein 1
11	S100-A1	Protein S100-A1
12	S100-A11	Protein S100-A11
13	S100-A16	Protein S100-A16
14	S100t	Protein S100
15	SHC1	SHC-transforming protein 1
16	SHE	SH2 domain-containing adapter protein E
17	SLC25A51	Solute carrier family 25 member 51
18	TLR1	Toll-like receptor 1
19	TLR19	Toll-like receptor 19
20	TRIM 35-27	Tripartite motif-containing 35-27
21	UBE2K	Ubiquitin-conjugating enzyme E2 K

Supplementary Table S2. NCBI accession numbers of TLR amino acid sequences used for the construction of phylogenetic tree

Sl	NCBI accession number	Name
1	XP_005262719.1	TLR1 *Homo sapiens*
2	XP_006503914.1	TLR1 *Mus musculus*
3	TWW55543.1	TLR1 *Takifugu flavidus*
4	ACT68332.1	TLR1 *Ctenopharyngodon idella*
5	ACV92063.1	TLR1 *Oncorhynchus mykiss*
6	XP_010895755.1	TLR1 *Esox lucius*
7	XP_032384491.1	TLR1 *Etheostoma spectabile*
8	XP_028445178.1	TLR1 *Perca flavescens*
9	XP_026213132.1	TLR1 *Anabas testudineus*
10	XP_031175177.1	TLR1 *Sander lucioperca*
11	APM84340.1	TLR1 *Dicentrarchus labrax*
12	XP_028271716.1	TLR1 *Parambassis ranga*
13	XP_022607278.1	TLR1 *Seriola dumerili*
14	AYM26735.1	TLR1 *Trachinotus ovatus*
15	XP_026186162.1	TLR1 *Mastacembelus armatus*
16	XP_031600531.1	TLR1 *Oreochromis aureus*
17	AKJ66261.1	TLR1 *Miichthys miiuy*

18	XP_027865028.1	TLR1 *Xiphophorus couchianus*
19	XP_019221940.1	TLR1 *Oreochromis niloticus*
20	XP_030252175.1	TLR1 *Sparus aurata*
21	XP_011478513.1	TLR1 *Oryzias latipes*
22	XP_026772609.1	TLR1 *Pangasianodon hypophthalmus*
23	AEI59662.1	TLR1 *Ictalurus punctatus*
24	AAI63271.1	TLR1 *Danio rerio*
25	XP_029587338.1 (TLR6)	TLR1 *Salmo trutta*
26	AUF71965.1	TLR19 *Ctenopharyngodon idella*
27	AEI59675.1	TLR19 *Ictalurus punctatus*
28	CDH93609.2	TLR19 *Salmo salar*
29	BAU98390.1	TLR19 *Cyprinus carpio*
30	APT35508.1	TLR19 *Megalobrama amblycephala*
31	NP_001352353.1	TLR19 *Danio rerio*
32	XP_029602002.1 (TLR12)	TLR19 *Salmo trutta*
33	XP_021414698.1	TLR13 Chr13 *Oncorhynchus mykiss*
34	XP_014049954.1	TLR13 Chrssa03 *Salmo salar*
35	XP_018974763.1	TLR13 Chr32 *Cyprinus carpio*
36	XP_026073895.1	TLR13 *Carassius auratus*

37	XP_009303038.2	TLR13 *Danio rerio*
38	KAA0715757.1	TLR13 *Triplophysa tibetana*
39	XP_015464101.2	TLR13 Chr19 *Astyanax mexicanus*
40	XP_024261195.1	TLR13 *Oncorhynchus tshawytscha*
41	XP_029513396.1	TLR13 *Oncorhynchus nerka*
42	XP_026654751.1	TLR13 *Zonotrichia albicollis*
43	XP_030825523.1	TLR13 *Camarhynchus parvulus*
44	XP_029631730.1	TLR13 Chr1 *Salmo trutta*
45	XP_030237431.1	TLR13 *Gadus morhua*
46	XP_030630757.1	TLR13 Chr5 *Chanos chanos*
47	AUO16779.1	TLR13 *Epinephelus coioides*
48	XP_026203040.1	TLR13 *Anabas testudineus*
49	XP_023250866.1	TLR13 *Seriolalalandi dorsalis*
50	XP_018939858.1	TLR13 Chr7 *Cyprinus carpio*
51	RXN21033.1	TLR13 *Labeo rohita*
52	XP_026155776.1	TLR13 *Mastacembelus armatus*
53	XP_031652369.1	TLR13 *Oncorhynchus kisutch*
54	XP_021264186.1	TLR13 *Numida meleagris*
55	XP_029547005.1	TLR13 Chr2 *Salmo trutta*

56	XP_015463807.2	TLR13 Chr12 *Astyanax mexicanus*
57	XP_019897826.1	TLR13 *Esox lucius*
58	XP_014005963.1	TLR13 Chrssa16 *Salmo salar*
59	XP_029612585.1	TLR13 Chr6 *Salmo trutta*
60	XP_018534808.1	TLR13 *Lates calcarifer*
61	XP_017579129.1	TLR13 *Pygocentrus nattereri*
62	XP_030634775.1	TLR13 Chr7 *Chanos chanos*
63	XP_026794334.1	TLR13 *Pangasianodon hypophthalmus*
64	ROI81776.1	TLR13 *Anabarilius grahami*
65	XP_029573484.1	TLR13 Chr27 *Salmo trutta*
66	XP_023844565.1	TLR13 *Salvelinus alpinus*
67	XP_021461145.1	TLR13 Chr6 *Oncorhynchus mykiss*
68	XP_031438389.1	TLR13 *Clupea harengus*
69	XP_007235237.2	TLR13 Chr8 *Astyanax mexicanus*
